# A Novel Weighted Ensemble Framework of Transformer and Deep Q-Network for ATP-Binding Site Prediction Using Protein Language Model Features

**DOI:** 10.3390/ijms27073097

**Published:** 2026-03-28

**Authors:** Jiazhi Song, Jingqing Jiang, Chenrui Zhang, Shuni Guo

**Affiliations:** College of Computer Science and Technology, Inner Mongolia Minzu University, Tongliao 028000, China; songjz671@nenu.edu.cn (J.S.); zhangcr@stu.ahtcm.edu.cn (C.Z.);

**Keywords:** protein–ATP binding sites prediction, transformer model, deep Q-network, ensemble learning, protein language model

## Abstract

Adenosine triphosphate (ATP) serves as a central energy currency and signaling molecule in cellular processes, with ATP-binding sites in proteins playing critical roles in enzymatic catalysis, signal transduction, and gene regulation. The accurate identification of ATP-binding sites is essential for understanding protein function mechanisms and facilitating drug discovery, enzyme engineering, and disease pathway analysis. In this study, we present a novel hybrid deep learning framework that synergizes heterogeneous learning paradigms based on protein sequence information for accurate ATP-binding site prediction. Our approach integrates two complementary base classifiers. One is a Transformer-based model, which leverages high-level contextual embeddings generated by Evolutionary Scale Modeling 2 (ESM-2), a state-of-the-art protein language model, combined with a local–global dual-attention mechanism that enables the model to simultaneously characterize short-segment and long-range contextual dependencies across the entire protein sequence. The other is a deep Q-network (DQN)-inspired classifier that achieves residue-level prediction as a sequential decision-making process. The final predictions are generated using a weighted ensemble strategy, where optimal weights are determined via cross-validations to leverage the strengths of both models. The prediction results on benchmark independent testing sets indicate that our method achieves satisfactory performance on key metrics. Beyond predictive efficacy, this work uncovers the intrinsic biological mechanisms underlying protein–ATP interactions, including the synergistic roles of local structural motifs and global conformational constraints, as well as family-specific binding patterns, endowing the research with substantial biological significance. The research in this work offers a deeper understanding of the protein–ligand recognition mechanisms and supportive efforts on large-scale functional annotations that are critical for system biology and drug target discovery.

## 1. Introduction

As the core energy carrier and signaling molecule in cells, adenosine triphosphate (ATP)’s specific binding to proteins plays an irreplaceable role in critical biological processes such as energy metabolism, enzyme catalysis, signal transduction, and gene expression regulation [1,2,3]. The specific regions of amino acid residues in proteins that can interact with ATP are termed as ATP-binding sites. Accurate analysis of their structure and function is not only a core prerequisite for understanding protein molecular mechanisms, but also provides a crucial theoretical basis for targeted drug design, enzyme engineering modification, and research on disease-related molecular pathways [4,5,6]. Although experimental techniques such as X-ray crystallography and nuclear magnetic resonance (NMR) imaging have successfully resolved the three-dimensional structures of some ATP-binding proteins, these methods suffer from limitations, including long experimental cycles, high costs, and strict requirements for sample purity, making them difficult to meet the demands of large-scale protein function annotation [7,8]. According to statistics, in recent years, the number of protein structures deposited in the Protein Data Bank (PDB) accounts for only a very small proportion of the total number of known sequence-based proteins, and, among these, structural data with clearly annotated ATP-binding sites are even scarcer [9]. Therefore, developing efficient and accurate computational prediction methods for ATP-binding sites based solely on protein sequence information has become a key breakthrough to compensate for the shortcomings of experimental techniques and advance proteomics research, which holds significant theoretical value and practical prospects.

In recent years, computational prediction methods for protein–ATP binding sites based on sequence information have developed rapidly, with continuous innovations in feature engineering, model architecture, and imbalance handling strategies. As early as 2018, Hu et al. [10] proposed the ATPbind predictor, which integrated sequence profiling features and template-based prediction results (S-SITEatp and TM-SITEatp) and adopted random under-sampling combined with a support vector machine (SVM) ensemble to address the data imbalance issue. Its sequence-only variant ATPseq achieved a Matthews Correlation Coefficient (MCC) of 0.639 and Area Under the Receiver Operating Characteristic Curve (AUC) of 0.878 on the ATP-41 testing set, outperforming most traditional sequence-based methods at that time. In 2020, Song et al. [11] pioneered the application of deep convolutional neural networks (CNNs) to this task, developing two network architectures: residual-inception-based and multi-inception-based predictors. By fusing position-specific scoring matrix (PSSM), predicted secondary structure, solvent accessibility, and one-hot encoding features, and using weighted cross-entropy to handle class imbalance, the ensemble model achieved an AUC of 0.896 and MCC of 0.635 on ATP-41, demonstrating the superior feature extraction capability of deep learning compared to traditional machine learning. In 2021, Song et al. [12] further optimized the prediction framework by integrating multi-IncepResNet, multi-Xception, and LightGBM into an ensemble model. This method adopted separate feature input strategies for different sequence features to reduce mutual interference and used weighted ensemble learning to fuse subclassifier outputs. On the ATP-41 testing set, it achieved an AUC of 0.902 and MCC of 0.642, setting a new benchmark for sequence-based prediction performance. In 2025, Gao et al. [13] proposed the S-DCNN method, which introduced the Synthetic Minority Over-sampling Technique (SMOTE) to balance the dataset while preserving sample information integrity. Novel feature parameters, including dihedral angles, energy values, and propensity factors, were incorporated, and hyperparameters of the deep convolutional neural network were optimized. On the ATP-41 testing set, it achieved an MCC of 0.5850 and an ACC of 96.78%, with an AUC of 0.8973, further enriching the feature types and data processing strategies for sequence-based prediction.

These studies have progressively advanced the performance of protein–ATP binding site prediction from traditional machine learning to deep learning and ensemble learning paradigms. However, the features used in these methods are traditional sequence features, which are manually designed and extracted from protein sequences (such as amino acid composition, physicochemical properties, and conservation scores). Their limitations have become increasingly prominent, meaning that these features are often constrained by prior biological knowledge, making it difficult to capture the implicit deep structural and functional associations in protein sequences [14,15]. Moreover, their ability to characterize complex patterns is limited, which leads to the poor generalization performance of models across datasets and complex scenarios [16]. With the development of deep learning technologies, large language models (LLMs) have provided a new paradigm for protein feature extraction and have demonstrated significant advantages in the field of predicting protein functional binding sites. For instance, a protein large language model has been introduced to the task of protein–DNA-binding site prediction, which effectively improved the prediction accuracy [17].

Therefore, in this study, we propose an ATP-binding site prediction framework that integrates Evolutionary Scale Modeling 2 (ESM-2) features with dual-model ensemble learning. Specifically, first, the ESM-2 protein language model [18] is used to extract deep features rich in biological semantics from the original sequences, instead of traditional manually designed features. Then, two core prediction models are constructed: one is a Transformer-based model containing innovative local–global dual-attention mechanism that captures long-range dependencies in the sequence through global attention while strengthening the fine interaction patterns near key residues through sliding window local attention and fuses the outputs of the two types of attention through dynamic weights; the other is a deep Q-network (DQN) model based on reinforcement learning, which optimizes action selection through experience replay and an ε-greedy strategy and strengthens the learning ability for hard samples using a reward mechanism. Finally, the prediction results of the two models are fused using ensemble learning to achieve performance complementarity. The experimental results show that the proposed method achieves an AUC of 0.9188 and an MCC of 0.6564 on the ATP-41 testing set and an AUC of 0.9353 and an MCC of 0.6625 on the ATP-17 testing set, which demonstrates the effectiveness of the proposed method in the ATP-binding site prediction task. The source code and datasets of the proposed method are available at https://github.com/tlsjz/ATPbindingEnsemble (accessed on 20 February 2026).

## 2. Results and Discussion

### 2.1. Performance Analysis of Local–Global Transformer Architecture

**Figure 1:**
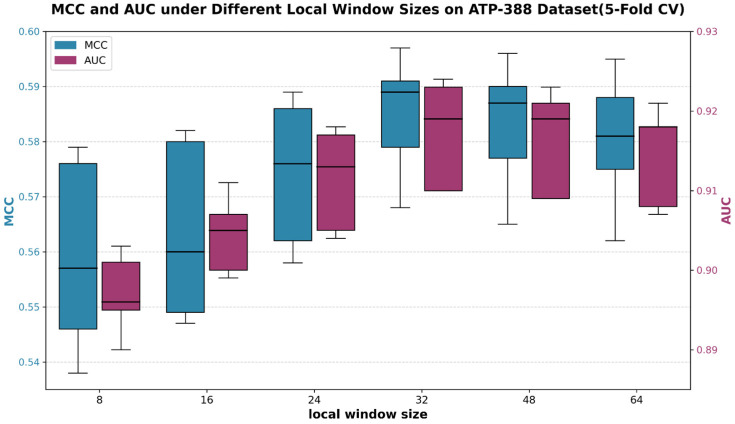
MCC and AUC comparison under different local window sizes over five-fold cross-validation on ATP-388.

A local–global dual-attention mechanism was constructed in the proposed Transformer-based model, which contained a local attention module that captured short-range residue feature interactions and a global attention module that captured long-range sequence dependencies. In the local attention module, the feature map was divided into local feature patches by the local window. Therefore, in order to determine the optimal local window size and elucidate its impact on prediction performance, we conducted five-fold cross-validation on ATP-388 with six candidate local window sizes (8, 16, 24, 32, 48, and 64) while fixing other hyperparameters. The MCC and AUC values under different local window sizes are shown in Figure 1. It can be found that the Transformer model with a local window size of 32 shows better performance with higher MCC and AUC values. For undersized local windows, only the very short and limited local dependencies within the amino acid sequence can be captured, which leads to the incomplete extraction of local features and the ineffective identification of key binding patterns. For oversized local windows, a substantial number of irrelevant amino acid residues are incorporated into local feature computation, which leads to the dilution of feature signals from core ATP-binding sites. Moreover, the increased window size elevates computational complexity, resulting in scattered attention on key interaction sites. Based on the above analysis, the optimal size of the local window in the Transformer model is 32.

Next, in order to validate the effectiveness of the proposed local–global dual-attention mechanism, we conducted a head-to-head comparison with a traditional Transformer model using five-fold cross-validation on the ATP-388 dataset. The quantitative results of the performance comparison are listed in Table 1.

**Figure 2:**
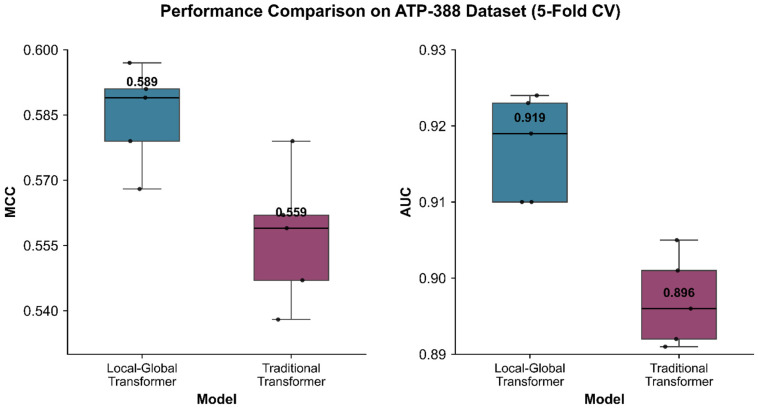
The MCC and AUC comparison between local–global transformer and traditional transformer over five-fold cross-validation on ATP-388.

The proposed Local–Global Transformer architecture outperformed the traditional Transformer model across all key metrics in five-fold cross-validation on the ATP-388 dataset. Specifically, for the dominant evaluation metrics, MCC and AUC, the local–global framework achieved an MCC of 0.585 and AUC of 0.917, with an accuracy of 0.969, sensitivity of 0.602, and specificity of 0.984. In contrast, the traditional Transformer yielded slightly lower performance metrics with an MCC of 0.557 and AUC of 0.897. Its other performance indicators were also marginally inferior. In order to make a more intuitive performance comparison, the MCC and AUC values between the Local–Global Transformer and traditional Transformer during five-fold cross-validation are shown in Figure 2.

The consistent performance gains of the Local–Global Transformer across all metrics highlight the advantages of its dual-attention design. The integration of local attention enables the model to capture fine-grained, short-range feature interactions that are critical for identifying subtle patterns in ATP-binding sites. This local contextual awareness complements the global attention module, which excels at modeling long-range dependencies, but may overlook localized discriminative features. By dynamically fusing these two attention outputs via learnable weights, the model achieves a more balanced representation of both local motifs and global structural contexts.

### 2.2. Synergistic Effect of Local and Global Attention: New Insights into ATP-Binding Molecular Mechanisms

To translate the technical design of the dual-attention mechanism into a deeper understanding of protein–ATP interactions, we constructed three variant models to dissect the independent and collaborative contributions of local and global sequence dependencies to ATP binding:

(1) Local-only Transformer: Only retains the local attention module (window size = 32), focusing exclusively on proximal residue interactions, which corresponds to local structural motifs of ATP-binding sites.
(2) Global-only Transformer: Only retains the global attention module, capturing solely distal residue dependencies, which reflects the global conformational constraints of proteins.
(3) Local–Global Transformer: The proposed dual-attention model, which is the dynamic fusion of local and global outputs.

**Figure 3:**
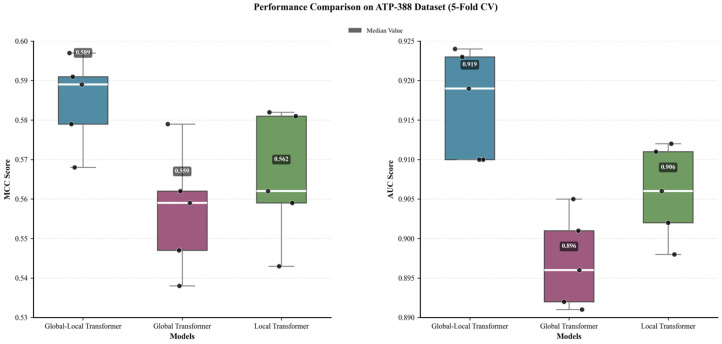
The MCC and AUC comparison of Local-only, Global-only, and Local–Global Transformer models over five-fold cross-validation on the ATP-388 dataset.

Five-fold cross-validation was performed on the ATP-388 dataset to ensure statistical robustness, and the performance of the three models was shown using the core metrics of MCC and AUC, which reflect the imbalanced classification performance and biological relevance in Figure 3 and Table 2.

As shown in Table 2, the Local-only Transformer achieved an MCC of 0.565 and an AUC of 0.906, while the Global-only Transformer yielded a lower MCC of 0.557 and an AUC of 0.897. Both single-attention models exhibited significant performance gaps compared to the Local–Global Transformer (MCC = 0.585, AUC = 0.917). Statistical analysis confirmed that the performance of the Local–Global Transformer was significantly superior to both single-attention models, indicating that neither proximal local interactions nor distal global dependencies alone are sufficient to fully capture ATP-binding characteristics.

To address the concern that five-fold cross-validation on the ATP-388 dataset may not fully reflect the actual arrangement of amino acid sequences in real scenarios, we further conducted a comprehensive performance comparison of the three models on the independent ATP-41 testing set. This testing set retains the complete natural sequence information of proteins, enabling a more authentic evaluation of the models’ ability to capture biological patterns. The performance metrics and corresponding confusion matrix results are summarized in Table 3.

It can be concluded from Table 3 that the Local–Global Transformer model maintains its outstanding performance compared to the single-Transformer model with an MCC of 0.625 and AUC of 0.916. However, beyond the metric increment, the differences in the number of true positive samples, false positive samples, and false negative samples are more meaningful. Compared to the single-Transformer model, the Local–Global Transformer model increased true positive samples by 25 and 44 while it reduced the false positive samples by 28 and 22. These coordinated changes can be translated to tangible practical value: for drug design, it expands the pool of potential target residues while filtering out irrelevant candidates, improving the efficiency of inhibitor development; for protein function inference, it ensures a complete and precise delineation of the binding pocket, laying a solid foundation for understanding the molecular mechanism of ATP–protein interactions; and for experimental validation, it reduces the burden of unnecessary assays by prioritizing biologically relevant residues, saving time and resources.

Moreover, we further analyzed the recognition rate for each type of amino acid in the three models’ prediction results. The Local-only Transformer mechanism exhibits notably higher recognition rates for Gly (82.3%) and Ser (78.5%) in ATP-binding sites. This is because Gly, as a core component of the P-loop/Walker A motif, relies on its small side chain to provide structural flexibility, enabling the motif to form a functional ATP-binding pocket by adapting to the spatial conformation of ATP’s phosphate groups. This flexibility is maintained through short-range interactions with adjacent residues, which are precisely the local sequence patterns captured by the local attention module. Similarly, Ser participates in ATP binding via short-range hydrogen bonds between its hydroxyl group and ATP’s phosphate moieties, with its binding specificity determined by immediate neighboring residues that are easily detected by local attention. However, the Local-only model performs poorly on Arg (60.7%), as Arg’s function in ATP binding is not only dependent on its local proximity to the binding pocket, but also on long-range global conformational constraints, such as maintaining the overall positive charge environment of the protein’s surface to complement ATP’s negatively charged phosphate groups and forming salt bridges with distal acidic residues to stabilize the binding pocket’s 3D structure. These global features are beyond the capture range of local attention.

In contrast, the Global-only Transformer mechanism achieves a significantly higher recognition rate for Arg (71.3%), as it can model long-range sequence dependencies and global charge distribution across the protein. Arg’s positive charge is often part of a large-scale electrostatic network on the protein surface, and its functional role in ATP binding requires coordination with distal residues to ensure spatial accessibility—features that the global attention module effectively captures. However, the Global-only model shows unsatisfactory recognition rates for Lys (70.2%) and Ser (70.3%). Lys, as another key component of the P-loop motif, forms direct electrostatic interactions with ATP’s phosphate groups through its ε-amino group, and its binding specificity is determined by its short-range motif context. Similarly, Ser’s hydrogen-bonding role is highly localized to the binding pocket’s immediate environment. The Global-only model, which prioritizes long-range dependencies, lacks sufficient sensitivity to these fine-grained local physicochemical interactions, leading to lower recognition accuracies for these residues.

The proposed Local–Global Transformer mechanism perfectly compensates for the limitations of both single-module models. By integrating the local motif-capturing capability and global conformational awareness, it achieves significant improvements in the recognition rates of core ATP-binding amino acids: Gly (91.5%), Arg (80.9%), Ser (86.3%), and Lys (83.6%). This enhancement arises from the model’s ability to perform “dual verification”: local attention identifies residues with motif-compatible short-range signals, while global attention validates their functional relevance by confirming conformational accessibility and global charge synergy. This mechanism ensures that functional residues are not missed due to insufficient local or global signal capture, and non-functional residues with pseudo-motif signals are effectively filtered out. These results indicate that while the overall performance gap between the Local-only and Global-only models is not overly pronounced, their recognition focuses are fundamentally different: the former excels at capturing local motif-dependent residues, while the latter specializes in global conformation-dependent residues. This leads to distinct prediction perspectives that are mutually complementary. The Local–Global attention mechanism effectively integrates these two perspectives, leveraging the strengths of each to overcome their individual limitations, ultimately achieving significant improvements in prediction performance and biological relevance.

By analyzing the amino acid recognition rate in prediction results from three models, some structural and functional features can be found to explain the functional values of important amino acids. For the amino acids Gly, Ser, and Lys, they play as core components of local motifs during binding process. Gly’s small hydrogen side chain provides the structural flexibility required to form a compact binding pocket that accommodates ATP’s phosphate groups, while Ser’s hydroxyl group forms short-range hydrogen bonds with ATP’s phosphate moieties. These specific characteristics make them irreplaceable roles in the P-loop/Walker A motif. For the amino acid Arg, its function in ATP binding is not limited to local charge complementarity, but also relies on long-range global conformational constraints. Arg residues often form salt bridges with distal acidic residues across protein domains to stabilize the 3D structure of the binding pocket, and their distribution contributes to the overall positive charge environment on the protein surface that enhances ATP recruitment. These findings are valuable for explaining the functional behavior of some important amino acids and underlying the molecular mechanism in the binding process.

The performance differences between the three models directly reveal key molecular features of protein–ATP interactions, providing quantitative computational evidence for a synergistic mechanism of ATP binding:

(1) Proximal local interactions are the core structural basis of ATP binding: The Local-only Transformer outperformed the Global-only Transformer, demonstrating that short-range physicochemical interactions between adjacent residues (e.g., hydrogen bonding, hydrophobic interactions in P-loop or Walker A/B motifs) form the primary recognition interface for ATP molecules. This aligns with classical biological findings that conserved local motifs are essential for ATP-binding [19], but our computational analysis further quantifies the contribution of these local interactions to binding site identification.
(2) Distal global dependencies provide critical conformational support: The Global-only Transformer achieved performance significantly higher than random guessing, revealing that long-range spatial arrangement of distal residues plays an indispensable role in maintaining the correct 3D conformation of ATP-binding pockets. Without this global conformational constraint, local core motifs may fail to form a functional binding interface, explaining why the Local-only Transformer cannot reach the performance of the dual-attention model.
(3) ATP-binding is a synergistic process of local recognition and global regulation: The optimal performance of the Local–Global Transformer confirms that ATP-binding site formation requires the collaboration of two complementary biological features: local structural motifs (providing specific binding interactions) and global conformational constraints (ensuring the spatial accessibility of binding sites). This synergistic mechanism resolves the long-standing ambiguity in computational biology regarding whether ATP-binding is dominated by local motifs or global structure; our results demonstrate that both are indispensable, with dynamic fusion of their contributions achieving the most accurate binding site prediction.

### 2.3. Impact of Contrastive Learning on Feature Discriminability and Biological Relevance of ATP-Binding Sites

To investigate the effect of the contrastive learning branch on feature discriminability and how it enhances the model’s capture of biologically meaningful signals, we first visualized the deep features (extracted before the classification layer) of the ATP-41 testing set using t-SNE [20] dimensionality reduction and then quantified the performance differences between the models with and without contrastive learning.

**Figure 4:**
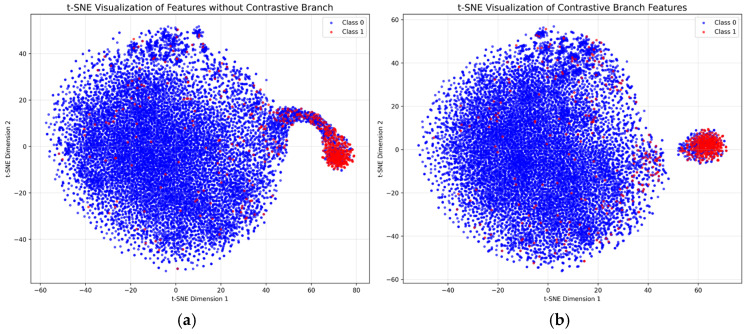
T-SNE distributions of deep features for models without (**a**) and with (**b**) the contrastive learning branch.

Figure 4a,b presents the t-SNE distributions of deep features for models without and with the contrastive learning branch, respectively (Class 0: non-ATP-binding residues; Class 1: ATP-binding residues). For the model without contrastive learning, Class 1 features (red points) are largely interspersed within the dense cluster of Class 0 features (blue points), with only a small subset of Class 1 residues forming a loose, partially overlapping sub-cluster. This indicates poor feature discriminability between the two classes, which stems from the biological nature of ATP-binding sites: ATP-binding residues are not only rare (accounting for only 3–5% of total residues in datasets), but also share partial physicochemical properties with non-binding residues (e.g., hydrophobicity, charge properties), leading to ambiguous feature boundaries in imbalanced data.

In contrast, after integrating the contrastive learning branch, the Class 1 features form a more compact and well-separated sub-cluster, with fewer red points mixed into the Class 0 cluster. This improvement directly aligns with the biological characteristics of ATP-binding sites: First, contrastive learning maximizes the similarity between augmented views of the same residue, forcing the model to focus on the conserved biological features of ATP-binding residues, such as the specific amino acid composition of P-loop motifs [21], the hydrophobic core of binding pockets [21], and the charge complementarity with ATP’s phosphate groups [22]. These conserved features are shared across ATP-binding residues, but vary from non-binding residues, explaining why Class 1 features become more clustered after contrastive learning. Second, by minimizing the similarity between features of different classes, contrastive learning amplifies the subtle biological differences between ATP-binding and non-binding residues. For example, ATP-binding residues often exhibit higher evolutionary conservation [23], and contrastive learning effectively enhances the representation of this conservation signal in features, distinguishing them from non-binding residues that lack such conservation.

Table 4 summarizes the performance of the two transformer models on the ATP-41 testing set. With the contrastive learning branch, the model achieved consistent improvements across all metrics, especially for MCC, which is the robust metric for imbalanced data. By integrating the contrastive learning branch, it improves from 0.601 to 0.625, and for AUC, which is threshold-independent discriminability, it increases from 0.897 to 0.916.

These performance gains are biologically meaningful for ATP-binding site prediction, as they address the core challenge of imbalanced data caused by the biological rarity of ATP-binding sites. Contrastive learning mitigates the model’s bias toward the majority non-binding residues by emphasizing conserved biological patterns of ATP-binding residues. This reduces false negatives (missed binding residues), which is critical for downstream experiments, for example, avoiding the omission of key residues in drug target design or enzyme engineering [24,25]. Moreover, the enhanced inter-class separability reduces false positives (misclassified non-binding residues), which aligns with the biological reality that ATP-binding sites are highly specific, which means that only residues with specific structural and physicochemical properties can interact with ATP’s adenine ring and phosphate groups [26].

Notably, the t-SNE visualization and performance metrics collectively demonstrate that contrastive learning does not merely optimize technical features, but rather refines the model’s capture of biologically meaningful signals, consistent with the evolutionary conservation and structural specificity of ATP-binding sites. This integration of technical design and biological insights advances our ability to distinguish functional ATP-binding residues from background sequences, highlighting the value of contrastive learning in bridging computational feature optimization and molecular mechanism understanding.

### 2.4. Weight Optimization of Ensemble Learning

To leverage the complementary advantages of two heterogeneous base classifiers, a weighted ensemble framework was constructed, and weight optimization was performed to maximize prediction performance for ATP-binding site identification. Five-fold cross-validation was conducted on the ATP-388 and ATP-227 datasets, with the sum of weights of the two base classifiers constrained to 1. The variations in key metrics, MCC and AUC, under different weight combinations were visualized to determine the optimal weight allocation.

**Figure 5:**
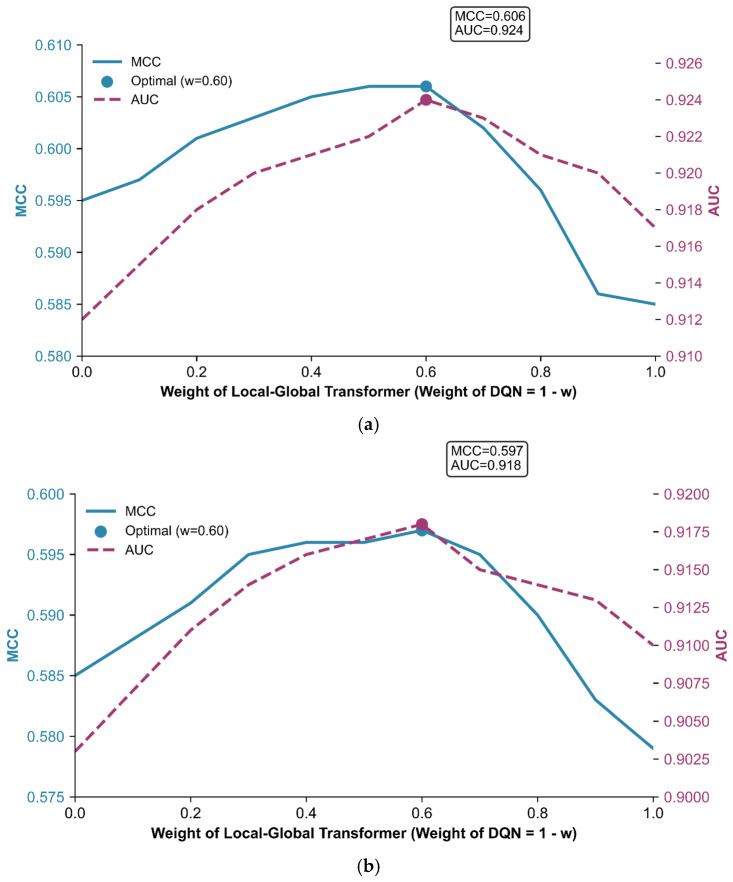
Prediction performance of ensemble model with varying weight distributions on ATP-388 (**a**) and ATP-227 (**b**) over five-fold cross-validation.

Figure 5a,b illustrates the performance trends of the ensemble model with varying weight distributions on the ATP-388 and ATP-227 datasets, respectively. A consistent pattern emerged across both datasets: as the weight of the local–global dual-attention Transformer classifier increased from 0 to 1, both MCC and AUC first rose to a peak and then gradually declined. Notably, the optimal weight combination converged to 0.6 for the Transformer classifier and 0.4 for the DQN classifier on both datasets, demonstrating the stability and generalizability of the weight optimization strategy. On the ATP-388 dataset, this optimal allocation achieved an MCC of 0.606 and an AUC of 0.924. On the ATP-227 dataset, the ensemble model yielded an MCC of 0.597 and an AUC of 0.918. In both cases, the ensemble performance was significantly superior to that of either base classifier alone.

The enhanced performance of the weighted ensemble model stems from the effective integration of the complementary strengths of the two base classifiers, as well as their synergistic mitigation of challenges posed by the inherent class imbalance of ATP-binding site datasets. First, the ensemble model complements feature modeling and decision-making paradigms. The Transformer classifier is specifically designed for imbalanced biological data. The local–global dual-attention mechanism in the Transformer classifier is capable of capturing fine-grained local sequence motifs and long-range global dependencies. Its integration of contrastive learning reinforces intra-class compactness and inter-class separability. In contrast, the DQN classifier, as a sequential decision-making process, leverages experience replay and an ε-greedy strategy to balance exploration and exploitation. Its reward-driven optimization enhances robustness to noise and fluctuations in sample distribution, which is particularly valuable for mitigating false positives in imbalanced scenarios. The optimal weight ratio (6:4) dynamically balances these strengths. Specifically, the Transformer classifier provides discriminative feature representations, while the DQN classifier refines decision boundaries to reduce misclassification of rare binding sites. Second, single classifiers often suffer from inherent limitations in imbalanced data. The Transformer classifier, despite its integration with contrastive learning, may still struggle with highly overlapping hard samples. Meanwhile, the DQN classifier, while robust to distribution shifts, may not effectively capture long-range dependencies in sequential data, which limits its ability to model the global context required for identifying ATP-binding patterns. The weighted ensemble addresses this by assigning a higher weight to the transformer classifier, which excels at identifying minority-class features while retaining the DQN classifier’s ability to suppress false positives. By dynamically adjusting their contributions, the ensemble model not only outperforms individual classifiers in key metrics, but also achieves superior adaptability to class imbalance. Therefore, the ensemble method proposed in this study provides a more reliable solution for ATP-binding site prediction.

### 2.5. Performance Comparison with State-of-the-Art Methods

**Figure 6:**
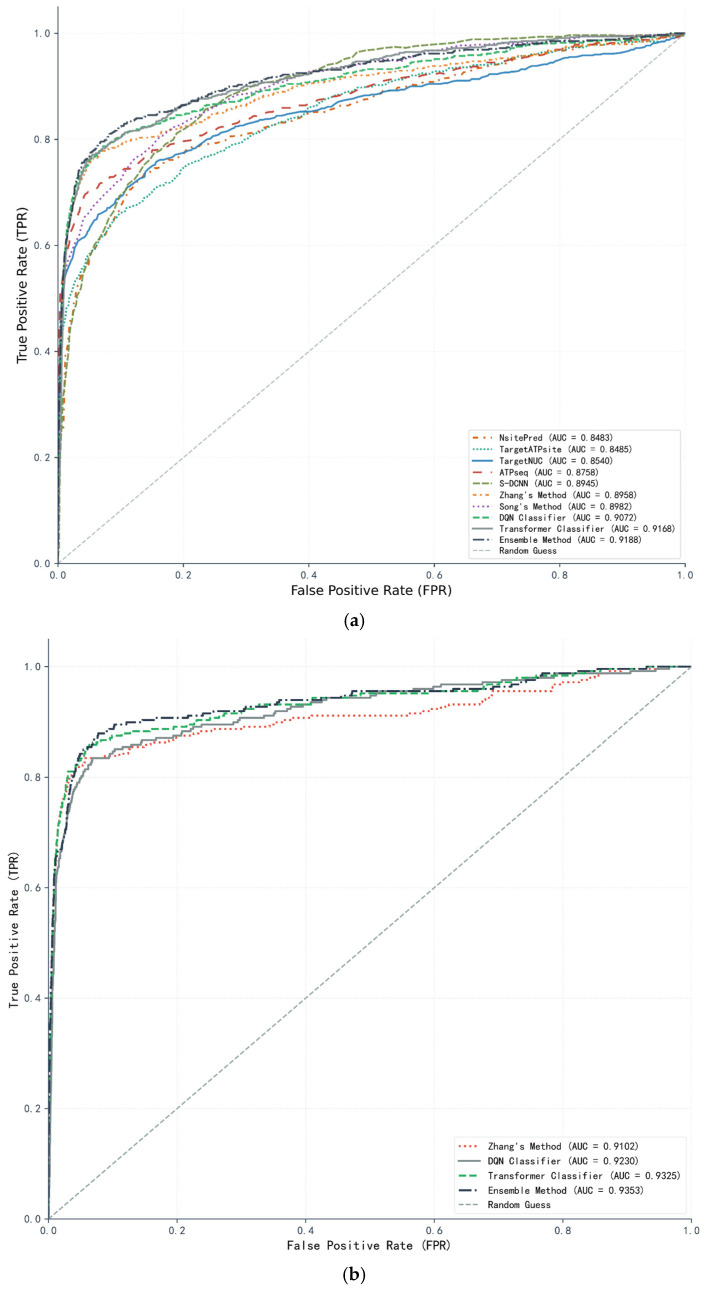
ROC curves of proposed method and some state-of-the-art prediction methods on ATP-41 (**a**) and ATP-17 (**b**) independent testing sets.

To comprehensively evaluate the effectiveness of the proposed methods for ATP-binding site prediction, we compared them with some state-of-the-art sequence-based prediction methods on two independent testing sets, ATP-41 and ATP-17. The performance metrics, including accuracy, sensitivity, specificity, Matthews Correlation Coefficient, and area under the Receiver Operating Characteristic (ROC) curve, were adopted for quantitative assessment, with the results summarized in Table 5 and Table 6 and the corresponding ROC curves illustrated in Figure 6a,b. The data in Table 5 and Table 6 were extracted from corresponding papers. However, some methods did not provide ROC curves of the ATP-17 testing set.

The comparative methods cover a spectrum of classical and contemporary approaches for nucleotide-binding site prediction. Some classical tools such as NsitePred [27], TargetATPsite [28], TargetNUC [29], and ATPseq [10] rely on handcrafted features and shallow classifiers such as support vector machines. Deep learning-based methods, including S-DCNN [13], leverage CNNs to capture local sequence patterns. The ensemble and hybrid methods encompass Song’s Method [12], which employs CNNs and LightGBM ensemble for ATP-binding site prediction, and Zhang’s Method [17], which was originally designed for protein-DNA binding site prediction and is adapted here by replacing input data with ATP-binding site features and fine-tuning hyperparameters for the classifier, integrated protein language model (PLM) features, and a pyramidal network with squeeze-and-excitation (SE) connections. Three proposed approaches are included: the local–global dual-attention Transformer classifier integrating contrastive learning, the DQN-based reinforcement learning classifier that models classification as a sequential decision-making process, and the weighted ensemble method that aggregates predictions of the two base classifiers with optimal weights determined via five-fold cross-validation.

As shown in Table 5 and Table 6, the proposed ensemble method consistently outperforms all comparative methods on both testing sets, which demonstrates its significant generalization capability. On the ATP-41 testing set, the ensemble method achieves an AUC of 0.919, MCC of 0.656, and sensitivity of 0.658, which are better than other state-of-the-art prediction methods, while the standalone Transformer and DQN classifiers also exhibit competitive performance that outperforms most comparative methods. Classical methods, such as NsitePred and TargetATPsite, show relatively lower performance, and this reflects the limitations of handcrafted features in capturing complex sequence patterns. On the ATP-17 testing set, the ensemble method maintains its leading position with an AUC of 0.935 and MCC of 0.663. The Transformer and DQN classifiers also achieve satisfactory performance, especially for sensitivity and AUC metrics. Across both testing sets, the proposed methods achieve substantial gains in AUC and MCC, which are critical for imbalanced data, indicating enhanced ability to identify rare ATP-binding residues without compromising specificity. Meanwhile, the ensemble method outperforms its individual base classifiers, which illustrates the complementary strengths of heterogeneous architectures.

The enhanced performance of the proposed methods stems from their targeted designs to address core challenges in ATP-binding site prediction, particularly class imbalance and complex sequence-based contextual dependencies. Unlike traditional CNN-based methods that focus solely on local sequence windows, such as S-DCNN, the Transformer classifier proposed in this study integrates a local–global dual-attention module to simultaneously model fine-grained local motifs and long-range global sequence dependencies. The dynamic fusion of local and global attention outputs, augmented by trainable positional encoding, enables comprehensive feature representation that exceeds the capabilities of single-scale feature extractors, which is particularly effective for ATP-binding sites where functional residues are often determined by both local physicochemical properties encoded in short sequence segments and global contextual patterns across the entire primary sequence. Complementing this, the Transformer classifier incorporates a contrastive learning branch that maximizes intra-class similarity and minimizes inter-class dissimilarity of sequence features, which is crucial for strengthening the compactness of minority-class features and expanding the feature gap between binding and non-binding residues to address model bias towards majority-class samples. The DQN classifier further enhances performance by modeling prediction as a sequential decision-making process. It leverages experience replay and an ε-greedy strategy to balance exploration and exploitation, combined with reward-driven optimization, which encourages the prioritization of rare binding residues while suppressing false positives. Its dynamic decision boundary adjustment, based on accumulated experience, makes it more robust to noise and distribution shifts in imbalanced data. Finally, the weighted ensemble framework aggregates the independent prediction outputs of the two base classifiers. The optimal weight allocation leverages their complementary strengths, which amplifies the contribution of the Transformer’s high-quality sequence feature representations and integrates the DQN’s refined prediction outcomes. The prediction results in performance gains that exceed those of individual base classifiers and homogeneous ensemble predictors highlight the value of integrating heterogeneous learning paradigms to address the multi-faceted challenges of ATP-binding site prediction.

### 2.6. Biological Relevance: Amino Acid Preferences and Family-Specific Performance

To enhance the biological relevance of our predictive model and advance the molecular-level understanding of protein–ATP interactions, we conducted a comprehensive validation encompassing three complementary analyses: amino acid composition consistency between true and predicted ATP-binding sites, recognition rate profiling of individual amino acids in functional binding sites, and family-specific performance evaluation across distinct ATP-binding protein classes.

**Figure 7:**
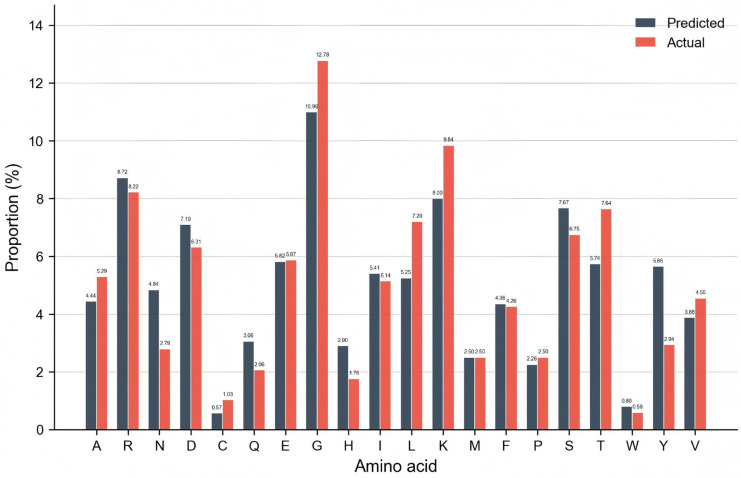
Comparison of amino acid composition between true and high confidence predicted ATP-binding sites in the ATP-41 testing set.

We first compared the amino acid composition of experimentally verified ATP-binding residues and high-confidence predicted residues in ATP-41 testing set. As illustrated in Figure 7, the amino acid distribution of the predicted sites exhibits a strong and statistically significant similarity to that of true sites, with a Pearson correlation coefficient of 0.87 (*p* < 0.001) and a mean absolute error (MAE) of only 1.18%. Notably, residues associated with canonical ATP-binding motifs—such as glycine (Gly, 10.99% predicted vs. 12.78% true), lysine (Lys, 8.00% vs. 9.84% true), and serine (Ser, 7.67% vs. 6.75% true), core components of the P-loop/Walker A motif [30]—are highly enriched in both groups, confirming the model’s ability to capture classical structural features of ATP-binding pockets. For hydrophobic residues (Leu, Ile, Val, Ala), their combined proportion is 22.18% in true sites and 23.28% in predicted sites, reflecting the consistent enrichment of hydrophobic residues that form the core to accommodate ATP’s adenine ring [13]. Basic residues (Lys, Arg) contribute 18.06% in true sites and 16.72% in predicted sites, aligning with their function in charge complementarity with ATP’s phosphate groups [31]. Furthermore, low-abundance residues in true sites (e.g., Cys, Trp, Tyr) are also rarely predicted, indicating that the model avoids false positives for biologically irrelevant residues and faithfully reflects the natural compositional constraints of functional ATP-binding sites.

To further characterize the biological interpretability of our model’s predictions, we calculated the recognition rate for each amino acid in true ATP-binding sites, defined as the percentage of true positive predictions for a given amino acid relative to its total count in the true binding sites. The results reveal clear patterns linked to the structural and functional roles of individual amino acids in ATP binding. Among these amino acids that form the protein sequence, glycine and lysine exhibit the highest recognition rates, with recognition rates of 94.25% and 85.05% respectively, reflecting their critical and conserved roles in ATP binding. Glycine’s small side chain provides the structural flexibility essential for the P-loop/Walker A motif, which forms the core of most ATP-binding pockets and is highly conserved across diverse ATP-binding proteins [32]. Lysine, another key component of the P-loop motif, contributes positive charge to interact electrostatically with ATP’s negatively charged phosphate groups [33]. In contrast, cysteine (42.86%) and proline (47.06%) show the lowest recognition rates, which can be attributed to their inherent biological properties: cysteine is prone to oxidation and disulfide bond formation, leading to structural variability in binding sites [34], while proline’s rigid pyrrolidine ring disrupts the local secondary structure and reduces sequence context conservation [35], increasing prediction difficulty. Most other residues, including hydrophobic residues (Ile, Leu, Val, ~60–65%) and polar residues (Tyr, ~75%), demonstrate moderate recognition rates, reflecting their context-dependent contributions to ATP binding, such as hydrophobic core formation or variable hydrogen bonding interactions.

Finally, we evaluated the model’s performance across three major ATP-binding protein families classified by Pfam [36] domain annotations, each representing distinct molecular mechanisms of ATP binding: kinases (PF00069, relying on local P-loop motifs and global kinase domain synergy), AAA+ ATPases (PF00004 subtype, dependent on global inter-subunit interactions in multimeric structures), and general P-loop NTPases (core PF00004, dominated by local motif interactions with global structural support). The family-specific performance metrics in Table 7 reveal that our model’s predictive power is closely tied to the structural and functional characteristics of each family. AAA+ ATPases achieve the highest performance, with an average MCC of 0.781 and an average AUC of 0.949, likely due to their strong reliance on global inter-subunit interactions, which our local–global dual-attention mechanism effectively captures. General P-loop NTPases show robust performance, with an average MCC of 0.726 and an average AUC of 0.918, consistent with their dependence on conserved local P-loop motifs that are prioritized by the model’s local attention module. Kinases exhibit relatively lower performance, a biologically meaningful observation that reflects their diverse substrate-specific domains that modify the context of ATP-binding pockets [37], increasing prediction complexity and highlighting a current bottleneck in kinase ATP-binding site prediction. This family-level analysis demonstrates that our model’s performance is not uniform, but varies in a biologically interpretable manner.

Moreover, the distinct predictive performance of the ensemble model across three ATP-binding protein families also uncovers intrinsic specificity of ATP-binding mechanisms unique to each family. For the AAA+ ATPases family, the functional binding pockets are formed only when multiple subunits assemble into oligomers, in which the distal residues from adjacent subunits contribute to the pocket’s 3D conformation and charge environment, while local P-loop motifs provide direct ATP contact. This dual requirement aligns perfectly with the ensemble model’s local–global dual-attention design, which explains the high performance for AAA+ ATPases. For the General P-Loop NTPases family, its binding specificity is dominated by conserved local P-loop motifs, which are highly conserved to form the primary recognition interface for ATP. Therefore, the local attention component in the prediction model plays a more important role, which effectively captures the key binding signals in local domains. The lowest performance on kinases reveals a unique binding specificity driven by family-specific substrate adaptation. Kinases retain the core P-loop motif, but modify its context via substrate-specific domains. These modifications introduce high structural heterogeneity, even among conserved P-loop residues. This diversification of binding specificity means that kinases lack universal local or global patterns that the model can reliably capture, explaining the lower predictive performance. These family-specific analyses reveal fundamental differences in ATP-binding specificity across protein families and deepen our understanding of how protein family evolution shapes ATP-binding mechanisms.

Collectively, the three complementary analyses—amino acid composition consistency, individual residue recognition rate profiling, and protein family-specific performance evaluation—provide multi-dimensional validation of the biological relevance and interpretability of our predictive model. The results demonstrate that our model’s performance is grounded in biologically meaningful patterns rather than statistical overfitting, bridging technical optimization with insights into protein–ATP interaction mechanisms and enhancing the study’s relevance to molecular science research.

### 2.7. Case Study on a Real Protein Sequence

**Figure 8:**
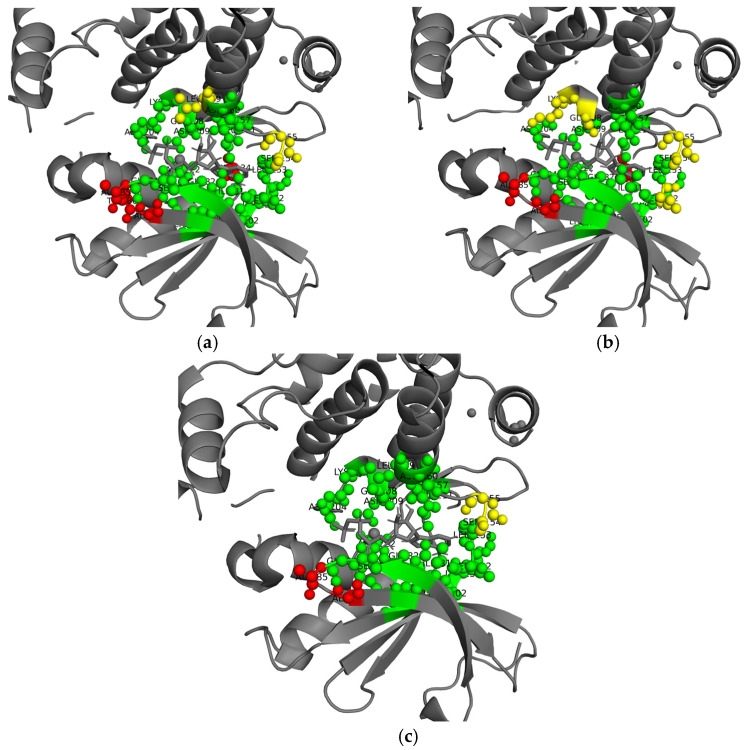
Prediction results from Transformer classifier (**a**), DQN classifier (**b**), and proposed ensemble method (**c**) on real protein with PDB ID of 5D9H_B.

To intuitively demonstrate the practical effectiveness of the proposed methods in real-world ATP-binding site prediction, a case study was conducted on the protein sequence 5D9H_B retrieved from the PDB database. The prediction results of the Transformer classifier, DQN classifier, and their weighted ensemble method were visualized using PyMOL (version 3.0.0) [38] software and are shown in Figure 8a–c. The green spheres represent true positive samples, which are correctly identified ATP binding sites. Red spheres denote false positive samples, which are non-binding residues misclassified as binding. Yellow spheres indicate false negative samples, which are ATP binding sites missed by the model.

For the Transformer classifier, 19 true positives (Ile17, Gly18, Ser19, Gly20, Val25, Ala38, Lys40, Met86, Lys87, Leu88, Leu89, Ser93, Asp96, Asp140, Lys142, Gly144, Asn145, Leu147, Asp158), four false positives (Ala21, Thr22, Ala23, Val70), and three false negatives (Ser90, Gly91, Leu95) were observed. The green TP spheres are concentrated within the functionally annotated ATP-binding pocket, with a clear enrichment of residues characteristic of the canonical P-loop/Walker A motif (Gly18, Gly20, Lys40, Gly144) and hydrophobic core residues (Ile17, Val25, Leu88, Leu89, Leu147). Meanwhile, the red FP spheres are moderately dispersed, primarily targeting small polar/aliphatic residues (Ala21, Thr22, Ala23, Val70) that share partial physicochemical properties with true binding residues but lack the specific sequence context required for ATP interaction. The yellow FN spheres (Ser90, Gly91, Leu95) indicate under-detection of conformationally variable binding residues, consistent with the challenge of distinguishing residues that occupy critical structural positions within the binding pocket, yet exhibit high local sequence similarity to non-binding regions.

The DQN classifier yielded 16 true positives (Ile17, Gly18, Ser19, Gly20, Val25, Ala38, Lys40, Met86, Lys87, Leu89, Ser93, Asp96, Asp140, Asn145, Leu147, Asp158), three false positives (Ala21, Ala23, Val70), and five false negatives (Leu88, Ser90, Gly91, Lys142, Gly144). While the DQN model exhibits a slightly lower false positive rate, its false negative count is increased, particularly affecting core motif residues (Lys142, Gly144) and hydrophobic residues (Leu88). This pattern reflects diminished sensitivity to subtle sequence conservation signals, which are critical for recognizing the conserved P-loop and hydrophobic core that stabilize ATP binding.

By contrast, the weighted ensemble method achieved 20 true positives (Ile17, Gly18, Ser19, Gly20, Val25, Ala38, Lys40, Met86, Lys87, Leu88, Leu89, Ser93, Leu95, Asp96, Asp140, Lys142, Gly144, Asn145, Leu147, Asp158), two false positives (Ala21, Ala23), and two false negatives (Ser90, Gly91). The green true positive spheres demonstrate enhanced spatial alignment with the experimentally validated binding domain, precisely covering all key P-loop residues (Gly18, Gly20, Lys40, Gly144) and hydrophobic core residues (Ile17, Val25, Leu88, Leu89, Leu95, Leu147). The red false positive spheres are further attenuated, eliminating non-specific predictions (e.g., Thr22, Val70), while the yellow false negative spheres are substantially minimized, retaining only two conformationally variable residues (Ser90, Gly91). This improvement is consistent with the synergistic fusion of the Transformer’s strong motif-capturing capability and the DQN’s decision robustness, enabling more reliable identification of both conserved and rare ATP-binding residues.

Notably, the ensemble method’s performance gains are biologically meaningful for practical applications: minimizing false positive samples reduces the experimental validation burden for non-functional residues, while increasing true positive samples enhances the coverage of functionally critical residues. For example, the precise identification of P-loop residues (Gly18, Gly20, Lys40, Gly144) is essential for drug target design, as mutations in these conserved positions often disrupt ATP hydrolysis and protein function [39]. The visual alignment of the ensemble’s predictions with the protein’s structural and functional context, combined with residue-level analysis, further validates its biological relevance.

## 3. Materials and Methods

### 3.1. Datasets

Two benchmark datasets are utilized in this study to comprehensively evaluate the performance of the proposed method.

The first dataset pair (ATP-227/ATP-17) is a classic benchmark proposed by Chen et al. [40] in 2011. ATP-227 comprises 227 non-redundant protein chains with pairwise sequence identity < 40%, where an amino acid residue is defined as an ATP-binding residue if the distance between any of its non-hydrogen atoms and those of the ATP molecule is less than 3.9 Å. This dataset contains 3393 ATP-binding residues and 80,409 non-binding residues, with a non-binding-to-binding residue ratio of 23.7. The independent testing set ATP-17 was constructed by selecting ATP-binding protein chains released after March 10, 2010, excluding any chains sharing >40% sequence identity with those in ATP-227 to avoid homology bias. It consists of 17 protein chains, including 248 binding residues and 6974 non-binding residues (ratio = 28.1). 

The second dataset pair (ATP-388/ATP-41) was proposed by Hu et al. [10] in 2018 to address the limitation of relatively small sample sizes in the classic dataset. It was derived from 2144 ATP-binding protein chains (PATP-2144) deposited in the PDB before November 5, 2016, with clear target annotations. Redundant sequences were removed using CD-hit software [41] with a sequence identity cutoff of 40%, and 429 non-redundant protein sequences were collected. These sequences were split into a training set (ATP-388) and an independent testing set (ATP-41): ATP-388 includes 388 protein chains deposited before 5 November 2014, containing 5657 binding residues and 142,086 non-binding residues (ratio = 25.1); and ATP-41 consists of 41 protein chains deposited after 5 November 2014, with 681 binding residues and 14,152 non-binding residues (ratio = 20.8). 

Detailed statistics of the four datasets, including the number of binding residues, non-binding residues, and their ratios, are summarized in Table 8.

### 3.2. The ESM-2 Protein Language Model

The ESM-2 protein language model, developed by Meta AI, represents a state-of-the-art tool for extracting biologically meaningful features from protein primary sequences. As a Transformer-based masked language model, ESM-2 is pre-trained on the UniRef50 database [42], encompassing 2.5 billion protein sequences, which enables it to capture deep evolutionary patterns, residue interaction dependencies, and functional motif information inherent in sequences. The model adopts a hierarchical Transformer architecture with functional differentiation across layers: the lower layers (1–11) focus on basic sequence pattern recognition (e.g., amino acid physicochemical property combinations and secondary structure elements), the middle layers (12–22) integrate domain-level interactions, and the upper layers (23–33) specialize in encoding functional sites (e.g., ligand-binding pockets) and evolutionary conservation signals. A key technical innovation is the integration of Rotary Position Embedding (RoPE), which effectively addresses performance degradation in long-sequence modeling by fusing absolute and relative position information, while dynamic token dropout and multi-precision inference further enhance its generalization ability and computational efficiency. For this study, the esm2_t33_650M_UR50D variant, which achieves a balance between predictive performance and computational cost for ATP-binding site prediction, was selected. It contains 33 transformer layers, 650 million parameters, and a 1280-dimensional hidden state.

### 3.3. Transformer-Based Prediction Model

After feature generation, we propose a Transformer-based [43] prediction model to identify ATP-binding sites based on ESM-2 features. The transformer-based model is built upon a novel Local–Global Transformer architecture, integrating contrastive learning and dynamic weighting mechanisms to address the challenges of imbalanced learning in protein–ATP binding site datasets. The model takes 1280-dimensional features generated by the ESM2 language model as input, where each feature vector corresponds to an amino acid residue in the protein sequence and outputs the probability of the residue being an ATP-binding site. The overall architecture consists of the following key components:

(1) Input processing and initial feature transformation

The input to the model is a 1280-dimensional feature vector for each amino acid residue, derived from the ESM2 pre-trained language model. These features are first reshaped into a sequence format (1 × 1280) to accommodate the Transformer-based processing pipeline. An initial dimensionality reduction step is applied using a dense layer with 384 hidden units, followed by layer normalization [44] and dropout [45] (rate = 0.15) to mitigate overfitting.

(2) Local–global dual-attention module

A core innovation of the model is the design of the local–global dual-attention module, which captures both fine-grained local contextual patterns and long-range global dependencies in protein sequences. The model first incorporates learnable position embeddings into the input features to encode the sequential order of amino acids. It employs an 8-head multi-head self-attention mechanism for global attention, which processes the entire sequence to model long-range interactions between residues. For local attention, aimed at capturing short-range interactions, the feature map is divided into local windows of size 32 through reshaping and transposition operations. The divided feature maps are processed by a separate multi-head attention mechanism and then projected back to the original dimension. The outputs of global and local attention are fused using a learnable weight parameter, which enables adaptive balancing of global and local contextual information.

(3) Contrastive learning branch

To address class imbalance in datasets, a contrastive learning [46] branch is integrated: features are projected to a 256-dimensional space with L2 normalization, and a temperature-scaled contrastive loss is applied to maximize intra-class similarity and minimize inter-class similarity. Numerical stabilization strategies (e.g., subtracting maximum similarity values, adding epsilon terms) are utilized to ensure robust training.

(4) Dynamic weight classification head

The final prediction is generated by a dynamic weight classification head: a sigmoid-activated dense layer produces a 384-dimensional weight vector to emphasize discriminative features via element-wise multiplication with latent features followed by a sigmoid layer to output binding site probabilities in the range [0, 1].

**Figure 9:**
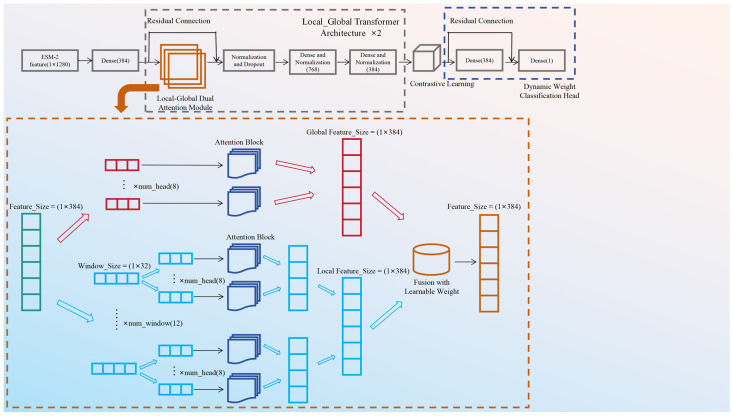
Overall architecture of transformer-based prediction model for ATP-binding sites.

The architecture of the proposed Transformer-based prediction model is shown in Figure 9.

The prediction model employs a hybrid loss function combining Focal Loss [47] and MCC penalty: Focal Loss (γ = 2.0, α = 0.2) downweights easy samples to focus on hard-to-classify residues, while an MCC penalty term (1 − max(MCC, 0), weighted 0.7×) optimizes for robust classification correlation in imbalanced data. Predictions and MCC values are clipped to ensure numerical stability. During the training process, the model is trained using the Adam optimizer with a cosine annealing learning rate schedule. A custom data generator handles imbalanced sampling by over-sampling binding sites and applies mild augmentation (Gaussian noise, random masking). The early-stop strategy is adopted to prevent over-fitting.

### 3.4. DQN Prediction Model Based on Reinforcement Learning

To further enhance the prediction performance, in addition to the transformer-based prediction model, we parallelly constructed a reinforcement learning-based [48] DQN model for ATP-binding site prediction. Unlike conventional supervised classifiers that optimize prediction accuracy via direct gradient descent on labeled data, our framework formulates the classification task as a sequential decision-making process where the agent learns an optimal policy by interacting with protein feature states. Each input protein sequence is represented as a fixed-dimensional feature vector *s* ∈ $R^{1280}$ extracted from the ESM-2 protein language model. The agent takes this feature as the state and performs an action *a* ∈ {0,1}, corresponding to the prediction of non-binding or binding, respectively. Action selection follows an ε-greedy strategy: accounting for the probability ε, a random action is chosen to encourage exploration; otherwise, the action with the highest estimated Q-value, $a={argmax}_{a}Q(s,a;\theta )$, is selected according to the current policy network parameterized by θ.

Upon making a prediction, the agent receives a reward signal designed to reflect classification correctness. Specifically, a positive reward of +1.0 is assigned for correct predictions, while incorrect ones receive a penalty of −0.5. To further guide long-term policy improvement, an additional terminal reward proportional to the batch-level accuracy is introduced at the end of each training episode. This hybrid reward mechanism balances immediate feedback with global performance trends.

The agent improves its policy through experience replay; transition tuples (*s*, *a*, *r*, *s’*, *done*) were stored in a replay buffer *R* to break temporal correlations between consecutive samples. Specifically, *s* denotes the current state, represented by a feature vector with a dimension of 1280 (derived from ESM-2); *a* corresponds to the action selected by the agent, which is a discrete class label (0 or 1) in this binary classification task; *r* is the immediate reward computed based on classification performance; *s’* represents the next state, which is set to be identical to *s* in this implementation; and *done* is a binary flag indicating whether the current state is terminal. Training was conducted by uniformly sampling mini-batches from *R* to minimize the temporal difference loss, defined as

(1) $$L\left(\theta \right)=E[{\left(r+\gamma (1-done\right)\cdot {max}_{a^{\prime }}\hat{Q}\left(s^{\prime },a^{\prime };\theta^{\prime }\right)-Q(s,a;\theta ))}^{2}]$$

where Q(s,a;θ) denotes the action-value function of the policy network with the parameter θ, which estimates the expected cumulative reward of taking action a in state s. $\hat{Q}\left(s^{\prime },a^{\prime };\theta^{\prime }\right)$ represents the target action-value function with delayed parameters $\theta^{\prime }$, where $\theta^{\prime }$ is updated every C episodes (set to 10 in this study) to stabilize training by decoupling the target from the policy network. The term ${max}_{a^{\prime }}\hat{Q}\left(s^{\prime },a^{\prime };\theta^{\prime }\right)$ computes the maximum expected future reward from the next state s’, discounted by a factor γ (0.95 in this work). The indicator (1−done) ensures that future rewards are only considered for non-terminal states.

During the training process, both the policy network and target network share an identical feedforward architecture: (1) an input layer accepting 1280-dimensional features; (2) two hidden layers with 512 and 256 neurons, respectively, each followed by ReLU activation and dropout regularization (rate = 0.3) to mitigate overfitting; and (3) an output layer with 2 neurons (corresponding to the two classes) that directly outputs Q-values without an activation function. All weights were initialized using Glorot uniform initialization to stabilize the training dynamics. The policy network was optimized using the Adam optimizer with a learning rate of 10–4, and gradient clipping (clipnorm = 1.0) was applied to prevent gradient explosion, thereby enhancing training stability. An ε-greedy strategy was adopted for action selection, where ε decayed from 1.0 to a minimum of 0.01 with a decay rate of 0.995, which balances exploration and exploitation. The training process of proposed deep Q-network for ATP-binding site is shown in Algorithm 1.

In order to further explore the potential of the DQN framework, we attempted to replace the original feedforward policy/target network with more complex architectures. However, the prediction performance did not achieve significant improvements. The reason for this might be that the core of the DQN framework lies in the reward-driven decision-making and experience replay mechanism, rather than the expressiveness of the network itself. For the binary classification task of ATP-binding site prediction, the original feedforward network is sufficiently expressive to model the mapping between feature states and Q-values. More complex architectures do not address the fundamental challenges of the task, but instead add computational overhead without complementary gains.
**Algorithm 1** Deep Q-Network(DQN) Training for ATP-Binding Site Prediction**Input**: Protein feature states $s\in R^{1280}$, labels $y\in \left\{0,1\right\}$; 1: Hyperparameters: discount γ, batch size *B*, episodes *E*, 2: exploration decay rate, target updata frequency *C***Output**: Trained policy network *Q*(*s*, *a*; θ), optimized prediction policy 3: Initialize policy network *Q*(*s*, *a*; θ) 4: Initialize target network $\hat{Q}$(*s*, *a*; $\theta^{\prime }$) with $\theta^{\prime }\leftarrow \theta$
 5: Initialize replay buffer *R* with capacity *M* 6: Set initial exploration rate ε=1.0, $\varepsilon_{min}=0.01$
 7: Optimizer ← Adam($lr=1\times 10^{-4}$, clipnorm=1.0) 8: **for** episode = 1 to *E* **do** 9:  $\varepsilon \leftarrow max\left(\varepsilon_{min},\varepsilon \times 0.995\right)$
 10: **for** each batch $\left(S,Y\right)$ in training set **do** 11:  **for** each state s∈S **do** 12:   **if**
rand()<ε **then** 13:    a← random action from $\left\{0,1\right\}$
 14:   **else** 15:    $a\leftarrow \arg{max}_{a}Q(s,a;\theta )$
 16:   **end if** 17:  **end for** 18:  $r_{immediate}\leftarrow +1.0$ if a=y, else −0.5
 19:  $r_{terminal}\leftarrow 2.0$ × batch accuracy onlyh on last batch 20:  $r_{total}=r_{immediate}+r_{terminal}$
 21:  done← True if last batch else False 22:  **for** each transition $\left(s,a,r_{total},s,done\right)$ **do** 23:   *R*.add$\left(s,a,r_{total},s,done\right)$
 24:  **end for** 25:  **if** $\left|R\right|\geq B$ **then** 26:   Sample mini-batch $\left\{\left(s_{j},a_{j},r_{j},s_{j}^{\prime },{done}_{j}\right)\right\}$ from *R*    // Compute target values using target network 27:   $q_{next}\leftarrow \hat{Q}\left(s_{j}^{\prime };\theta^{\prime }\right)$
 28:   $q_{target}\leftarrow r_{j}+\left(1-{done}_{j}\right)\times \gamma \times {max}_{a}q_{next}$
    // Update policy network via gradient descent 29:   $q_{current}\leftarrow Q(s_{j};\theta )$
 30:   $q_{pred}\leftarrow \sum_{a}q_{current}\times ⟦a=a_{j}⟧$
 31:   Compute loss: $L=\frac{1}{B}\sum {\left(q_{pred}-q_{target}\right)}^{2}$
 32:   Update θ using gradients from Adam optimizer 33:  **end if**
 34:  **end for** 35:  **if** episode mod C==0 **then** 36:   $\theta^{\prime }\leftarrow \theta$
 37:  **end if** 38: **end for** 39: **Return** optimized policy network Q(s,a;θ)


### 3.5. Overall Architecture of Proposed Prediction Method

**Figure 10:**
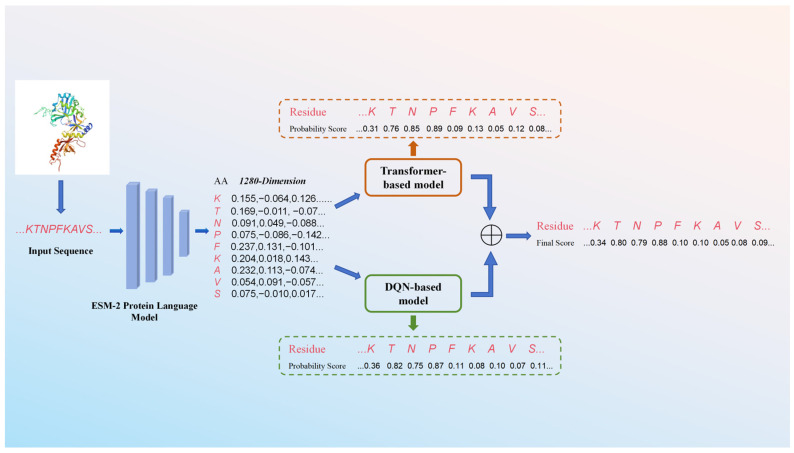
Workflow of proposed prediction method for ATP-binding site.

This study proposes an integrated framework for ATP-binding site prediction, which combines deep language model feature extraction, dual-classifier modeling, and ensemble learning to improve prediction robustness. The workflow of the framework, from protein sequence input to final probability generation, is shown in Figure 10.

First, the input protein sequence (in FASTA format) is processed by the ESM-2 protein language model, which encodes each amino acid residue into a 1280-dimensional deep feature vector. These features capture evolutionary conservation, residue physicochemical properties, and latent functional patterns inherent in the sequence, providing a high-quality representation for downstream classification tasks.

Next, the 1280-dimensional residue-level features are fed into two parallel classification models: (1) a Transformer-based model with a local–global dual-attention module, which models both short-range residue interactions and long-range sequence dependencies; and (2) a DQN-based reinforcement learning model, which frames the prediction task as a sequential decision-making process to prioritize biologically meaningful patterns via dynamic reward feedback. Each model independently outputs a probability score for each residue, which indicates the likelihood of it being an ATP-binding site.

Finally, an ensemble strategy is applied to fuse the outputs of the two models: the probability scores from the Transformer and DQN models are weighted and summed to generate the final prediction probability for each residue. This weighted fusion leverages the complementary strengths of the two models and consequently enhances the overall prediction performance on imbalanced biological sequence data.

### 3.6. Evaluation Metrics

Given the severe class imbalance (a small number of ATP-binding sites versus a large number of non-binding sites) in the ATP-binding site prediction task, a comprehensive set of evaluation metrics was adopted to objectively assess the performance of the proposed framework. These metrics include both threshold-dependent indicators (Accuracy (ACC), Sensitivity (Sen), Specificity (Spe), Matthews Correlation Coefficient (MCC)) and a threshold-independent indicator (Area Under ROC Curve (AUC)), ensuring a holistic evaluation of classification performance across different dimensions. All threshold-dependent metrics are derived from the confusion matrix, including four crucial components: the number of true positive samples (TP), the number of true negative samples (TN), the number of false positive samples (FP), and the number of false negative samples (FN) in the prediction result.

Accuracy measures the overall correctness of predictions, calculated as the ratio of correctly classified samples to the total number of samples:

(2) $$ACC=\frac{TP+TN}{TP+TN+FP+FN}$$

However, ACC is less reliable for imbalanced data due to its susceptibility to the dominant class, and is thus complemented by other metrics. Sensitivity evaluates the model’s ability to identify actual ATP-binding sites, emphasizing the minimization of false negatives:

(3) $$Sensitivity=\frac{TP}{TP+FN}$$

Specificity quantifies the model’s ability to correctly classify non-binding sites, reflecting its resistance to false positives:

(4) $$Specificity=\frac{TN}{TN+FP}$$

The Matthews Correlation Coefficient is widely recognized as the most robust metric for imbalanced classification, as it integrates all four confusion matrix variables and ranges from −1 to 1 (a value of 1 indicates perfect classification, 0 denotes random classification, and −1 represents complete misclassification):

(5) $$MCC=\frac{TP\times TN-FP\times FN}{\sqrt{\left(TP+FP)(TP+FN)(TN+FP)(TN+FN\right)}}$$

Consistent with previous ATP-binding site prediction studies, the optimal classification threshold for all threshold-dependent metrics was determined by maximizing the MCC value, which balances the trade-off between Sen and Spe while accounting for class imbalance.

As a threshold-independent metric, the AUC is computed as the area under the ROC curve, which plots the TPR (Sen) against the FPR (false positive rate) across all possible classification thresholds. The AUC reflects the model’s ability to distinguish between binding and non-binding sites regardless of the threshold setting, with a value of 1 indicating perfect discrimination and 0.5 corresponding to random guessing. It is particularly valuable for comparing model performance on imbalanced data, as it is not biased by class distribution.

## 4. Conclusions

Accurate identification of protein–ATP binding sites is critical for deciphering cellular mechanisms and advancing drug discovery. Existing computational methods often face challenges related to insufficient feature representation and severe class imbalance in binding site data. To address these limitations, this study proposes a novel weighted ensemble framework that integrates a Transformer-based classifier and a DQN-based reinforcement learning model and leverages ESM-2-derived sequence features to synergize their complementary strengths for enhanced prediction performance. The proposed method achieves enhanced performance on two benchmark datasets and demonstrates better performance than state-of-the-art sequence-based predictors across key metrics. The satisfactory prediction performance of the proposed method indicates its outstanding capability in protein–ATP binding site identification, which is crucial for bridging the gap between the exponential growth of uncharacterized protein sequences and the scarcity of experimentally validated binding site annotations. Meanwhile, this study uncovers fundamental biological rules underlying protein–ATP interactions, moving beyond mere technical optimization to deliver mechanistic insights. First, the proposed method delivers the key biological insight that the binding of protein and ATP molecules relies on the synergy of local structural motifs and global conformational constraints. Second, Gly, Lys, Ser, and Arg are functionally irreplaceable amino acids due to their conserved roles in motif formation, electrostatic interaction, and global charge balance. Finally, we uncover family-specific ATP-binding specificity patterns across major protein families. This study establishes a reliable and efficient computational method for ATP-binding site prediction, which not only advances the integration of protein language models and heterogeneous learning paradigms in structural biology and bioinformatics, but also deepens mechanistic insights into protein–ATP interactions to inform drug target design, enzyme engineering, and functional annotation of uncharacterized proteins. Future work will focus on integrating multi-source features and developing adaptive ensemble strategies to enhance generalization across diverse protein families.

## Figures and Tables

**Table 1:** Performance comparison with Local–Global Transformer and traditional transformer with five-fold cross-validation on ATP-388.

	Accuracy	Sensitivity	Specificity	MCC	AUC
Traditional transformer	0.967	0.596	0.981	0.557	0.897
Local–Global Transformer	0.969	0.602	0.984	0.585	0.917

**Table 2:** Performance comparison of Local-only, Global-only, and Local–Global Transformer models over five-fold cross-validation on the ATP-388 dataset.

	Accuracy	Sensitivity	Specificity	MCC	AUC
Global Transformer	0.967	0.596	0.981	0.557	0.897
Local Transformer	0.968	0.597	0.981	0.565	0.906
Local–Global Transformer	0.969	0.602	0.984	0.585	0.917

**Table 3:** Performance comparison of Local-only, Global-only, and Local–Global Transformer models on the ATP-41 testing dataset.

	Accuracy	Sensitivity	Specificity	MCC	AUC	TP	TN	FP	FN
Global Transformer	0.963	0.556	0.983	0.566	0.876	379	13,958	237	302
Local Transformer	0.964	0.584	0.982	0.584	0.889	398	13,964	243	283
Local–Global Transformer	0.968	0.621	0.985	0.625	0.916	423	13,936	215	258

**Table 4:** Performance comparison between transformer models with and without contrastive learning on ATP-41 testing set.

	Accuracy	Sensitivity	Specificity	MCC	AUC
Without contrastive learning	0.966	0.598	0.983	0.601	0.897
With contrastive learning	0.968	0.621	0.985	0.625	0.916

**Table 5:** Performance comparison with other sequence-based prediction methods on ATP-41 testing set (methods are ranked according to AUC).

Method	Accuracy	Sensitivity	Specificity	MCC	AUC
NsitePred	0.954	0.467	0.977	0.456	0.852
TargetATPsite	0.968	0.413	0.995	0.559	0.853
TargetNUC	0.972	0.469	0.997	0.627	0.856
ATPseq	0.972	0.545	0.993	0.639	0.878
S-DCNN	0.968	0.510	0.990	0.585	0.897
Zhang’s Method	0.970	0.596	0.988	0.633	0.896
Song’s Method	0.973	0.497	0.996	0.642	0.902
DQN Classifier	0.967	0.626	0.986	0.639	0.907
Transformer Classifier	0.968	0.621	0.985	0.625	0.916
Ensemble Method	0.971	0.658	0.986	0.656	0.919

**Table 6:** Performance comparison with other sequence-based prediction methods on ATP-17 testing set (methods are ranked according to AUC).

Method	Accuracy	Sensitivity	Specificity	MCC	AUC
NsitePred	0.967	0.460	0.985	0.476	0.875
TargetATPsite	0.972	0.458	0.991	0.530	0.882
TargetNUC	0.975	0.516	0.992	0.584	--
Zhang’s Method	0.976	0.709	0.985	0.657	0.910
Song’s Method	0.978	0.589	0.992	0.639	0.925
DQN Classifier	0.978	0.656	0.989	0.661	0.923
Transformer Classifier	0.975	0.625	0.987	0.618	0.932
Ensemble Method	0.978	0.657	0.990	0.663	0.935

**Table 7:** Prediction performance across three ATP-binding protein families in ATP-41 testing set.

Protein Family	Accuracy	Sensitivity	Specificity	MCC	AUC
Kinases (PF00069)	0.559	0.855	0.564	0.412	0.580
ATPases (AAA+; PF00004 subtype)	0.972	0.861	0.979	0.781	0.949
General NTPases (Core P-loop NTPase; PF00004)	0.962	0.760	0.974	0.726	0.918

**Table 8:** Statistical characteristics of the datasets used in this study.

Dataset	Number of Protein Chains	Number of ATP-Binding Residues	Number of Non-Binding Residues	Non-Binding/Binding Ratio
ATP-227	227	3393	80,409	23.7
ATP-17	17	248	6974	28.1
ATP-388	388	5657	142,086	25.1
ATP-41	41	681	14,152	20.8

## Data Availability

The source code and datasets of proposed method is available at https://github.com/tlsjz/ATPbindingEnsemble (accessed on 20 February 2026).
